# Whole body resistance training on functional outcomes of patients with Stage 4 or 5 chronic kidney disease: A systematic review

**DOI:** 10.14814/phy2.16151

**Published:** 2024-08-12

**Authors:** Salma Abrahim, Alexandra P. Steele, Jennifer Voth, Joan C. Krepinsky, Matthew B. Lanktree, Thomas J. Hawke

**Affiliations:** ^1^ Department of Pathology and Molecular Medicine McMaster University Hamilton Ontario Canada; ^2^ Research and Evaluation Services Department, Hôtel‐Dieu Grace Healthcare Windsor Ontario Canada; ^3^ Division of Nephrology, St. Joseph Healthcare Hamilton and Department of Medicine McMaster University Hamilton Ontario Canada

**Keywords:** exercise training, kidney disease, chronic, physical fitness, resistance exercise, systematic reviews, weight‐lifting exercise

## Abstract

Chronic kidney disease (CKD) causes skeletal muscle wasting, resulting in reduced function and inability to live independently. This systematic review critically appraised the scientific literature regarding the effects of full‐body resistance training on clinically‐relevant functional capacity measures in CKD. The study population included studies of people with Stage 4 or 5 CKD and a mean age of 40+ years old. Eight databases were searched for eligible studies: Pubmed, Embase, Cochrane, CINAHL, Scopus, Web of Science, MEDLINE, and AGELINE. MeSH terms and keyword combinations were used for screening following the PRISMA conduct. Inclusion criteria were based on PICO principles and no date of publication filter was applied. The intervention was training 2 days/week of structured resistance exercises using major upper and lower muscle groups. Minimum intervention period was 7 weeks. Comparison groups maintained their habitual activity without structured exercise training. Outcome measures of interest were: 6‐min walk test, grip strength, timed up‐and‐go test, and sit‐to‐stand. Eight randomized controlled trials and one nonequivalent comparison‐group study fulfilled the inclusion criteria and underwent data extraction. All studies were of hemodialysis patients. The evidence indicates that full‐body resistance exercise significantly improved grip strength, timed up and go and sit to stand tests; metrics associated with enhanced quality and quantity of life.

## 1 INTRODUCTION

Chronic kidney disease (CKD) is often a progressive disease, leading to the loss of kidney function over time and resulting in end‐stage kidney disease which requires dialysis or transplantation to sustain life (Ronai & Sorace, 2008; Webster et al., 2017). The prevalence of CKD has increased by 30% within the past two decades, with an estimated global incidence of >800 million people (Bikbov et al.*,* 2020; Kovesdy, 2022). The most common nongenetic causes of CKD are diabetes and high blood pressure (Webster et al., 2017) while autosomal dominant polycystic kidney disease (ADPKD) is the most common form of inherited kidney disease (Bergmann et al., 2018; Mahboob et al., 2024). The risk of CKD is increased in individuals older than 65, and is more common in Native Americans, African‐Americans and individuals who are obese and have a history of autoimmune diseases (Webster et al., 2017).

The progression of CKD forms a continuum ranging from early stage (Stage 1) to late stage (Stage 5; kidney failure requiring dialysis or transplantation) (Stevens & Levin, 2013; Webster et al., 2017). Clinically, these stages are defined by the estimated glomerular filtration rate (eGFR) (Jin et al., 2008; Stevens & Levin, 2013). In general, persons with CKD are asymptomatic until Stage 4 or 5 when signs and symptoms such as fatigue, weight loss and weakness may develop (Arora, 2024). Even with maintenance dialysis, muscle wasting is a common complication of CKD which leads to frailty and associated declines in the capacity for independent living and quality of life (Moorthi & Avin, 2017). Importantly, it is associated with increased risk of morbidity and mortality (Cheng et al., 2022; Moorthi & Avin, 2017).

Numerous etiologies for the loss of muscle health have been reported including: dysregulated protein turnover, recommended reductions in protein intake, anemia, metabolic acidosis, insulin resistance, inflammation, reduced physical activity and the catabolic effects of dialysis (Cheng et al., 2022). While the relationship between skeletal muscle health and kidney function was not always a clinical consideration, the strong correlations between skeletal muscle mass, mortality and major adverse cardiovascular events is bringing skeletal muscle health to the forefront in CKD care. In fact, the incidence of muscle atrophy in CKD patients who have not undergone dialysis was reported at 30% with a hazard ratio of death at 2.62 (Carrero et al., 2008). These muscle health and mortality outcomes worsen when dialysis treatment is required, with incidence of muscle atrophy reported at 39% of the population and a hazard ratio of death of 3.04 (Carrero et al., 2008).

Clearly, interventions to improve both the quality and quantity of life for those with CKD is urgently needed and focusing on restoration of skeletal muscle health could be an important and tangible target. Resistance training, which is a type of exercise that requires a muscle group to contract against external force, is associated with an increase in the size and/or strength of skeletal muscle in both healthy and diseased populations (McLeod et al., 2019; Rhee & Kalantar‐Zadeh, 2014). The purpose of this study was to synthesize the evidence for the benefits of whole body resistance training on clinically‐relevant metrics of muscle function in Stage 4 and 5 CKD patients.

## 2 METHODS

The goal of this systematic review was to consolidate our current knowledge on the effects of resistance exercise training on four clinically relevant muscle function outcomes. The systematic review protocol has been registered with the International Platform of Registered Systematic Review and Meta‐analysis Protocols (INPLASY): DOI:10.37766/inplasy2024.5.0083. See Table 1 for description of test and the clinical relevance of the test. Through measuring these outcomes pre‐ and post‐resistance training, this systematic review can be used to examine the scope of functional improvement in persons with CKD.

**TABLE 1:** Brief description of the clinically relevant functional tests included in this review.

Functional test	Brief description of test	Clinically‐relevant outcome
6‐min walk (6MWT)	Total distance walked without assistance over a 6‐min period	Indicator of cardiopulmonary and musculoskeletal response to exercise (Bellet et al., 2013; Karanth & Awad, 2017; Trudelle‐Jackson & Jackson, 2018)
Sit to stand	Number of “full stands from a seated position” (without using hands) completed in 30 s. May also be measured as the number of full stands from seated position in a fixed period of time.	Measure of lower extremity strength, balance, disability and falls risk (Bellet et al., 2013; Karanth & Awad, 2017; Trudelle‐Jackson & Jackson, 2018)
Grip strength	Subject maximally squeezes a hand grip dynamometer three times with each hand. Maximal grip of the three repetitions for each hand is recorded.	Surrogate measure of overall muscle strength (and lower grip strength has been associated with all‐cause mortality and disability) (Prasitsiriphon & Pothisiri, 2018; Puhan et al., 2008)
Timed up & go	Time it takes for a seated subject to stand, walk a set distance (e.g., 3 meters), walk back and sit back down.	A metric of lower extremity function, mobility and fall risk (Whitney et al., 2005; Witherspoon et al., 2018)

### 2.1 Eligibility criteria

Inclusion criteria were based on the PICO (participant, intervention, comparison and outcome) principles. The study population included studies of adults with a mean age of 40 years of age and older with Stage 4 or 5 CKD (with and without dialysis). The intervention was resistance training following Canadian Society for Exercise Physiology (CSEP) guidelines of 2 days/week of structured resistance exercises that use major muscle groups (24Hour Movement Guidelines, 2021). Moreover, the minimum intervention period was 7 weeks, as a previous meta‐analysis has shown optimal improvements in health outcomes from resistance training at this intervention duration (Ashton et al., 2020). The comparison group was participants who maintained their usual physical activity habits and were not undergoing exercise training. The outcome measures were: 6MWT, grip strength, timed up‐and‐go test, and sit‐to‐stand. No filter was applied with respect to the date of publication.

Articles were excluded if they were opinion‐based or if they were published in a language other than English. They were also excluded if the mean age of the study population was <40, had exercise training that was less than 7 weeks long, had resistance training that was <2 days/week, had no control group, had no confirmation that resistance training was included in the methodology or were not human‐based studies. A mean age of <40 years old was chosen as an exclusion criteria in an effort to focus on the patient population that is most likely to suffer from declines in the clinically‐relevant metrics that were chosen as outcome measures. The full inclusion and exclusion criteria can be found below in Table 2.

**TABLE 2:** Detailed inclusion and exclusion criteria.

Inclusion criteria	Exclusion criteria
(a) The study population had a mean age of 40 years and older (b) The population was individuals clinically diagnosed with stage 4 or 5 CKD with and without dialysis (i) Stage 4 CKD GFR = 15–29 mL/min/1.73 m^2^ (ii) Stage 5 CKD GFR = <15 mL/min/1.73 m^2^ (c) The intervention was resistance exercise training with a minimum of 2 days of training/week during the intervention period (d) The resistance exercise training RET included upper and lower body training. (e) The intervention period was at minimum 7 weeks long (f) The control group (comparison group) was sedentary or recreationally active and maintained their regular activity patterns (with no training). The recreationally active group did not regularly include resistance exercise training in their activity patterns. (g) The outcome was a performance based test, including 6MWT, Grip Strength, Timed Up‐and‐Go test, or Sit‐to‐Stand (h) The article was peer reviewed and published in English	(a) It was an opinion paper (b) It was published in a language other than English (c) The mean age of the study population was under 40 years old (d) The exercise training was less than 7 weeks long (e) Resistance training was less than 2 days/week (f) No control group was included (g) No confirmation of compliance of regular resistance exercise training was included in the methodology

The articles returned from each database search were compiled and any duplicates were removed before undergoing title and abstract screening.

### 2.2 Search strategy

This review followed the Preferred Reporting Items for Systematic Reviews and Meta‐Analyses (PRISMA) conduct (Figure S1) (Page et al., 2021). The framework consisted of defining a PICO research question, creating a set of inclusion/exclusion criteria and search terms, conducting a search through databases, selecting articles for inclusion through title and abstract screening followed by full text screening. Screening was undertaken independently by two review authors (SA, APS).

**FIGURE 1:**
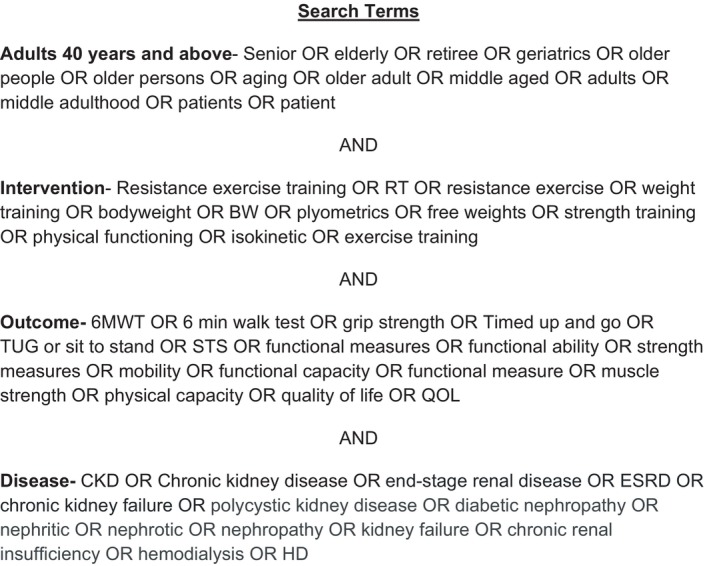
Full list of search terms.

The following 8 databases were searched for eligible studies: Pubmed, Embase, Cochrane, CINAHL, Scopus, Web of Science, MEDLINE, and AGELINE. The database search included articles published on or before December 31, 2023. MeSH terms and keyword combinations were used for screening articles. Search terms specific for population, intervention, outcome, and disease, as detailed in Figure 1 were used. Duplicate data were then identified and removed using Rayyan, a reference management website (Ouzzani et al., 2016).

### 2.3 Data extraction

**FIGURE 2:**
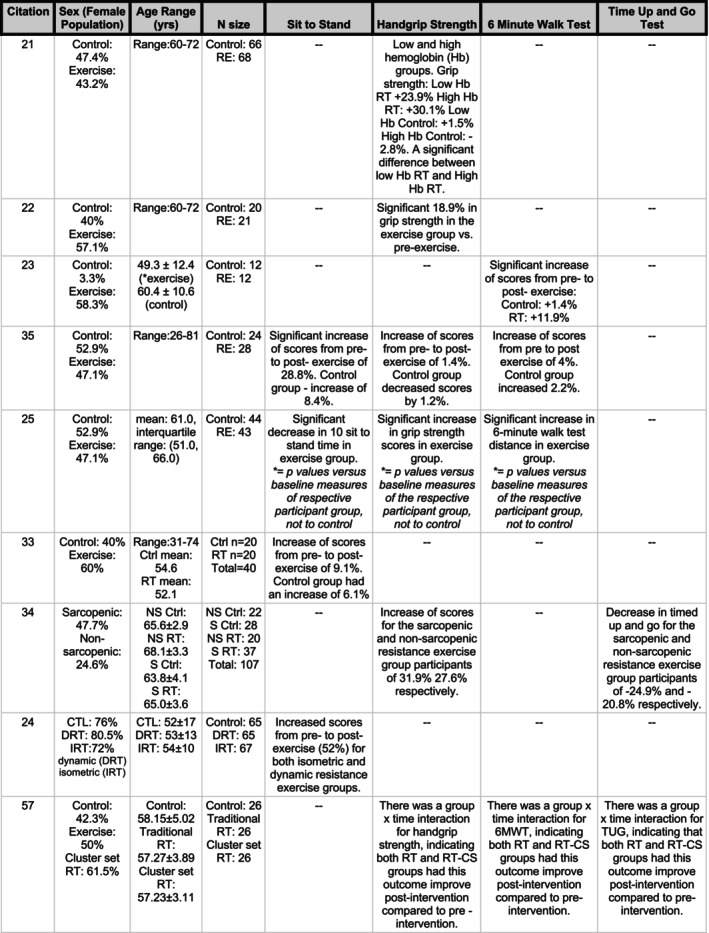
Data extraction table with information on number of participants, age range of sample, % of female participants, exercise intervention details with outcome information and results.

Data extraction was performed through Google Spreadsheet to identify characteristics of each study, including first author, country, publication year, sample size, stage of CKD, outcomes measured, description of exercise intervention, age range, and main results for each outcome. A full data extraction table can be seen in Figure 2. Data were extracted independently by two review authors (SA, APS) and for missing and unclear information, study authors were contacted for additional details.

### 2.4 Methodological quality assessment

The risk of bias for the included studies was assessed via the Cochrane risk‐of‐bias tool (RoB 2) (Sterne et al., 2019). The RoB 2 is the recommended and standard tool to asses bias and it includes the following domains: bias arising from the randomization process, bias due to deviations from intended interventions, bias due to missing outcome data, bias in measurement of the outcome and bias in selection of the reported result (Sterne et al., 2019). For each domain, the risk of bias is stated, and is facilitated by an algorithm that maps the preceding responses to the signaling questions asked to a proposed judgment. Response options to the signaling questions are yes, probably yes, probably no, no, and no information (Sterne et al., 2019). Once the questions are answered, the risk of bias judgment is assigned using one of the following three levels in each domain: low risk of bias, some concerns or high risk of bias (Sterne et al., 2019). Two authors (SA, APS) scored each of the included articles independently and any discrepancies were resolved through discussion until a consensus was achieved.

The Cochrane RoB assessment tool was chosen for its standardization and consistency. Developed by the Cochrane Collaboration, the Cochrane RoB tool benefits from a high level of trust and credibility within the scientific community. Furthermore, though originally designed for randomized control trials, the principles underlying the Cochrane RoB tool are easily adapted for systematic reviews.

### 2.5 Calculation of effect sizes

Although a systematic review methodology was undertaken for the present study, effect sizes were calculated for each outcome reviewed, if aggregate data was available from primary source, to provide a comprehensive examination of the data. Effect sizes (dppc) for mean differences with groups with unequal group sizes within pre‐post‐control research designs were computed based on Carlson & Schmidt, (1999) and as recommended by Morris (2008). Data were entered into an effect size calculator to compute dppc for each study under review. All effect sizes were obtained using the calculator found on www.psychometrica.de/effect_size.html#cohenb. Effect sizes of ≥0.2, ≥0.5, and ≥0.8 reflected small, medium, and large effect sizes, respectively Lakens (2013).

## 3 RESULTS

### 3.1 Included studies

The PRISMA flowchart of the selection process can be found in Figure S1. With the search strategy, a total of 32,799 articles were identified. After removing duplicate articles, 26,587 articles were analyzed through title and abstract screening, and 120 articles were eligible for full text review. The total number of articles that fulfilled the inclusion criteria and were included in the data extraction were 8 randomized controlled trials and 1 nonequivalent comparison‐group study (da Silva et al., 2021; de Castro et al., 2023; Dong et al., 2019; Gadelha et al., 2021; Martins do Valle et al., 2020; Rosa et al., 2018, 2021; Song & Sohng, 2012; Zhang et al., 2020).

### 3.2 Study characteristics

The age range of participants in the included studies was between 26 and 81 years (Rosa et al., 2018). Study size ranged between 24 (Martins do Valle et al., 2020) to 197 (Rosa et al., 2018) participants (total of 760 subjects). Most of the articles did not state demographic information such as ethnicity, but there were no significant differences between groups for the article that did state the prevalence of ethnicity groups (Rosa et al., 2018). There were no significant differences in sex across groups in all included articles. All participants were hemodialysis patients, with three interventions occurring during dialysis sessions (Martins do Valle et al., 2020; Rosa et al., 2021; Zhang et al., 2020), 5 occurring pre‐dialysis (da Silva et al., 2021; de Castro et al., 2023; Dong et al., 2019; Gadelha et al., 2021; Song & Sohng, 2012) and one study in which exercises occurred both before and during dialysis (Rosa et al., 2018). All outcomes were measured at baseline and at the end of the intervention period. Five studies were 12 weeks in duration (Dong et al., 2019; Martins do Valle et al., 2020; Rosa et al., 2021; Song & Sohng, 2012; Zhang et al., 2020) and four were 24 weeks in duration (da Silva et al., 2021; de Castro et al., 2023; Gadelha et al., 2021; Rosa et al., 2018). Exercise sessions for five studies were supervised by a certified professional, two were not supervised while one study did not state whether supervision occurred (da Silva et al., 2021). Exercise sessions were performed two to three times per week, while intensity was reported for five studies and ranged from 5 to 13 measured on the Borg Rating of Perceived Exertion (RPE) scale (da Silva et al., 2021; de Castro et al., 2023; Martins do Valle et al., 2020; Rosa et al., 2021; Zhang et al., 2020).

### 3.3 Risk of bias for included studies

All nine articles were judged to have some risk of bias concern, as they did not document the full details for the study to have minimal bias (da Silva et al., 2021; de Castro et al., 2023; Dong et al., 2019; Gadelha et al., 2021; Martins do Valle et al., 2020; Rosa et al., 2018, 2021; Song & Sohng, 2012; Zhang et al., 2020).

### 3.4 Exercise‐related adverse events

Participating in physical exercise may increase the risk of adverse events. Zhang and colleagues looked at the differences in expected and reported adverse events in the exercise and control group (Zhang et al., 2020). The expected events reported in the respective exercise and control groups, were palpitations (1 vs. 1), hypotension (3 vs. 3) and musculoskeletal events (cramps: 3 vs. 0 and muscle soreness: 4 vs. 1) (Zhang et al., 2020). There were no life‐threating adverse events that were observed during the study, and there were no significant differences in the incidence of adverse events between the control and exercise groups (Zhang et al., 2020).

### 3.5 Exercise‐related effects on functional outcomes

Eight of the nine articles assessed handgrip strength with a hydraulic hand dynamometer, with the average of attempts taken in kilograms of force (kgf) (Table 3). Six out of the eight articles found a significant (intermediate to large effect size) improvement in handgrip strength scores with training (Mean delta ±SD; Control: 0.14 ± 1.14 kgf versus Training: 6.87 ± 3.06 kgf) (da Silva et al., 2021; Dong et al., 2019; Gadelha et al., 2021; Rosa et al., 2021; Song & Sohng, 2012; Zhang et al., 2020). There were two articles that did not find a change in handgrip strength following resistance exercise training (Control delta: 0.58 ± 1.44 kgf versus Training delta: 1.63 ± 1.09 kgf) (de Castro et al., 2023; Rosa et al., 2018).

**TABLE 3:** Summary of Included Articles.

Study	Outcome measure	Pre‐control	Post‐control	Pre‐intervention	Post‐intervention	*p*‐value	Effect size *d*_*ppc*_ Morris (2008)
da Silva et al. (2021)	Hand grip	20.0 (5.1)	19.7 (5.5)	21.1 (4.3)	27.1 (4.3)	0.0001	1.333***
Dong et al. (2019)	Hand grip	20.99 (6.05)	21. 34 (6.16)	22.23 (5.27)	26.03 (3.85)	0.006	0.597**
Zhang et al. (2020)	Hand grip	22.47 (7.01)	22.03 (7.09)	25.71 (8.48)	26.57 (8.43)	0.170 (control); 0.03 (intervention)	0.166
Song and Sohng (2012)	Hand grip	26.2 (10.2)	27.8 (11.8)	26.3 (8.5)	28.7 (9.0)	0.465	0.084
Gadelha et al. (2021)—sarcopenia only group vs. control	Hand grip	20.7 (4.7)	20.28 (1.61)	20.8 (5.7)	27.44 (3.59)	< 0.0001	1.317***
Gadelha et al. (2021)—non‐sarcopenia group vs. control	Hand grip	20.6 (6.2)	19.65 (1.93)	21.4 (4.5)	27.3 (2.51)	< 0.0001	1.231***
Rosa et al. (2021)—resistance training vs. control	Hand grip	24 (8)	26 (5)	23 (6)	35 (4)	<0.0001	1.269***
Rosa et al. (2021)—isometric training vs. control	Hand grip	24 (8)	26 (5)	25 (5)	38 (7)	<0.0001	1.633***
Rosa et al. (2018)	Hand grip	59.21 (20.66)	58.52 (18.19)	65.71 (23.27)	66.61 (22.22)	0.213	0.071
de Castro et al. (2023)—control vs. traditional RT	Handgrip	21.07 (6.20)	20.42 (6.11)	21.61 (5.20)	26.26 (5.45)	<0.05	0.912***
de Castro et al. (2023)—control vs. cluster RT	Handgrip	21.07 (6.20)	20.42 (6.11)	20.61 (4.49)	27.57 (3.71)	<0.05	1.385***
Rosa et al. (2018)	6 min walk test	452.65 (169.19)	469.42 (162.93)	506.13 (130.34)	526.45 (126.15)	0.277	0.023
Rosa et al. (2018)	Sit to stand	10.88 (3.04)	11.79 (2.93)	11.79 (3.47)	15.18 (6.07)	0.015	0.745**
Zhang et al. (2020)	6 min walk test	373.57 (89.63)	373.21 (91.30)	406.54 (85.61)	409.49 (88.27)	0.026 (intervention); 0.665 (control)	0.037
Zhang et al. (2020)	Sit to stand	25.80 (2.06)	26.40 (2.59)	25.20 (23.40, 26.70)	23.80 (23.10, 25.70)	<0.001 (intervention); 0.028 (control)	Noting that Zhang reports intervention sit to stand in medians and the control group as means^a^
Gadelha et al. (2021)—sarcopenia only group vs. control	Timed up and go	8.5 (2.2)	9.58 (1.44)	7.8 (1.9)	5.86 (0.56)	< 0.0001	1.467***
Gadelha et al. (2021)—non‐sarcopenia group vs. control	Timed up and go	9.2 (1.9)	9.87 (1.29)	10.0 (1.4)	7.92 (0.71)	< 0.0001	1.605***
de Castro et al. (2023)—control vs. traditional RT	Time up and go	17.03 (4.09)	17.64 (3.70)	17.16 (3.77)	14.48 (3.10)	<0.05	−0.824***
de Castro et al. (2023)—control vs. cluster RT	Time up and go	17.03 (4.09)	17.64 (3.70)	17.88 (3.65)	13.63 (2.84)	<0.05	−1.235***
Martins do Valle et al. (2020)	6 min walk test	487.9 (63.4)	494.8 (66.9)	408.5 (161.3)	457.3 (155.6)	0.04	0.33*
de Castro et al. (2023)—control vs. traditional RT	6 min walk test	407.03 (100.37)	413.69 (104.68)	447.96 (93.25)	545.53 (92.1)	<0.05	0.924***
de Castro et al. (2023)—control vs. cluster RT	6 min walk test	407.03 (100.37)	413.69 (104.68)	435.57 (91.83)	565.96 (86.56)	<0.05	1.267***

*Note*: Effect sizes were not computed based on available data from primary source. Reported unable to compute effect size based on data reported in primary source.

*** Reflects a large effect size.

** Reflects a medium effect size.

* Reflects a small effect size.

^a^ Intervention baseline and follow‐up sit‐to‐stand measurements are originally reported as medians (IQR), whereas control group measurements are reported as means ±SD.

Four articles looked at the impact of resistance training on the 6MWT (Table 3) (de Castro et al., 2023). Three of four articles found significant improvement in the 6MWT but the reported data are variable with two of these studies having notable (small and large) effect sizes (Mean delta ±SD; Control: 11.83 ± 6.98 m versus Training: 34.56 ± 20.14 m) (Zhang et al., 2020). The fourth article did not find a significant difference in the distance participants walked in 6 min post‐training (delta; Control: −0.36 m versus Training: 2.95 m) (Rosa et al., 2018).

Two articles investigated the impact of resistance training on the timed up‐and‐go test (de Castro et al., 2023; Gadelha et al., 2021). A significant improvement in time to perform this activity after the resistance training intervention was noted with this being a large effect size for both articles (Table 3) (de Castro et al., 2023; Gadelha et al., 2021).

Four studies explored the effect of resistance training on the sit‐to‐stand test (de Castro et al., 2023; Gadelha et al., 2021; Rosa et al., 2018; Zhang et al., 2020). Both studies found a significant improvement in this outcome. The first article by Zhang and colleagues (Zhang et al., 2020) saw a reduction in time for participants to perform 10 sessions of sit‐to‐stand (presented as median values; Control: pre: 25.80 sec, post: 26.40 sec. versus Training pre: 25.2 sec, post: 23.8 sec). Similarly, the study by Rosa et al (Rosa et al., 2018) saw an increase in the number of sit‐to‐stand sessions performed by participants in a 30 s period (Control: pre: 10.88, post: 11.79 versus Training pre: 11.79, post: 15.18; see Table 3).

### 3.6 Quality of the evidence

This systematic review included eight randomized controlled trials and 1 nonequivalent comparison‐group study (*n* = 760). However, all the included studies had limitations in their methodology that can reduce their validity. Only two of the included studies stated that outcome assessors were blinded (Song & Sohng, 2012; Zhang et al., 2020). The outcome assessors in the other seven studies were either not blinded or blinded was not reported, which can possibly bias the data collection process. Additionally, the different studies had a range of exercise intensities, durations and exercise types used for resistance training. For example, two of the studies presented had intervention periods for 12 and 24 weeks, with one being predialytic exercise and the other intradialytic exercise, and each had their own weight progression over the study period (da Silva et al., 2021; Dong et al., 2019).

These methodological differences could contribute to the variability of the observed changes in the measured outcome of grip strength. Taking these limitations into account, it would be important to interpret the conclusions of this systematic review with caution.

## 4 DISCUSSION

Physical activity and exercise are important aspects to preventing and treating chronic diseases, such as obesity and Type 2 diabetes mellitus as it increases health‐span and longevity (Chow et al., 2022). In addition, with the diagnosis of a chronic disease, treatment and management are more successful when exercise is implemented (Chow et al., 2022). Although there are non‐modifiable disease risk factors, modifiable factors such as regular exercise can significantly reduce an individual's risk for disease or mitigate the progression of co‐morbidities (Morris, 2008).

While pharmacological interventions can treat symptoms of disease, exercise works holistically to cause biological systems to function optimally (Chow et al., 2022). Those with CKD exhibit elevated risk of cardiovascular events in all stages, with a marked increase in the later stages of the disease (Jankowski et al., 2021). In fact, cardiovascular rather than end‐stage kidney disease (Stage 5) is the leading cause of death in this high‐risk population (Denic et al., 2016; Jankowski et al., 2021). Exercise training interventions not only improve muscle health outcomes for those with CKD, but may also positively affect several cardiovascular outcomes, at least in part through improving myocardial strength and lowering systolic blood pressure (Chow et al., 2022).

This systematic review has shown that resistance training is associated with improving clinically relevant, functional outcomes in Stage 4 and 5 CKD patients undergoing dialysis, a population prone to loss of muscle mass and strength (wasting). Importantly, no serious adverse events were reported in any of the exercise training intervention groups in any of the 9 studies that met the criteria to be included herein. One study specifically reported adverse outcomes as a secondary outcome of interest (Zhang et al., 2020). The researchers noted no life‐threatening adverse events were observed and of the adverse events reported (muscle soreness, hypotension, palpitations) no significant differences between exercising and control groups was observed. It is also important to appreciate that Gadelha et al. (Gadelha et al., 2021) undertook resistance training in CKD patients (65 ± 4 years) with and without sarcopenia. Subjects were randomly assigned into four groups: sarcopenic resistance training, non‐sarcopenic resistance training, sarcopenic control, and non‐sarcopenic control. As the coexistence of CKD and sarcopenia are strongly linked to mortality, the authors also followed death events of their participants over a 5‐year follow‐up. The overall death rate was 25.2%. It was observed that the proportion of deaths was higher for sarcopenic subjects (Control *n* = 36% vs. resistance trained *n* = 30%) compared to non‐sarcopenic subjects (Control *n* = 18% vs. resistance trained *n* = 10%). Importantly, mortality was significantly and consistently lower in those who had been included in the resistance training group. Taken together, these studies suggest that resistance training is not only safe for those with CKD undergoing dialysis (regardless of whether sarcopenia is present), but is likely to reduce 5‐year mortality.

The 4 functional metrics (hand grip strength, 6 min walk test, sit‐to‐stand, and timed up‐and‐go) were selected for their previously well‐established correlations to clinically important outcomes (including morbidity, mortality, fall risk and quality of life). Extensive literature has documented that a decline in hand grip strength is associated with increased length of hospital stays and higher risk of mortality (Prasitsiriphon & Pothisiri, 2018). These associations have been observed in the general population and in specific patient groups such as those with cardiovascular diseases and cancer (Prasitsiriphon & Pothisiri, 2018). In a systematic review by Chen and colleagues, as measuring hand grip strength is non‐invasive and can be easily measured by assessors, it has been widely recommended as a clinical means to stratify a patient's risk of mortality (Prasitsiriphon & Pothisiri, 2018). Despite the bulk of evidence supporting an increase in hand grip strength with resistance exercise training, Rosa et al. (2018) did not observe a significant improvement in handgrip strength after the exercise intervention (Rosa et al., 2018). These authors attributed the lack of improvement in grip strength to inclusion of only three upper body muscle groups (biceps, shoulders, and back) and low adherence, with exercises scheduled to occur before the dialysis session (Rosa et al., 2018). Furthermore, upper body exercises in hemodialysis patients can be difficult due to presence of the dialysis access in the upper limb, restricting exercise to only one limb during the dialysis session in those with a fistula or graft (Rosa et al., 2018). Clearly the efficacy of resistance exercise to improve grip strength is supported in most studies, but additional studies are needed to verify the feasibility and safety of upper body exercises during dialysis treatment.

The 6MWT reflects the submaximal level of exertion needed to perform daily physical activities, thereby assessing the functional capacity of patients (Bučar Pajek et al., 2016). It has been shown to be a highly reliable and validated measure of fatigability in muscle diseases and is also a predictor of mortality (Karanth & Awad, 2017; Witherspoon et al., 2018). Akin to the results for hand‐grip strength, Rosa and colleagues found no significant improvements in the 6MWT distance, contradicting findings in two other studies (Martins do Valle et al., 2020; Zhang et al., 2020). This result may be due to differences in intensity of the resistance exercise intervention between studies, as the two out of the three studies that reported improvements in 6MWT distance had set a higher intensity for their resistance exercise intervention (Martins do Valle et al., 2020, Zhang et al., 2020). Specifically, two studies that found improvement in 6MWT had one resistance exercise session during dialysis in which the weight was adjusted to allow the participant to perform a maximum of 12 repetitions of each exercise (Martins do Valle et al., 2020; Zhang et al., 2020). In contrast, the study by Rosa et al. had two shorter training sessions, one before dialysis and one during dialysis, in which the participant performed 15 to 20 repetitions of each exercise (Rosa et al., 2018). While predicting a relationship between a change in 6MWT and a clinical outcome (e.g., mortality) is not a 1:1 ratio (as numerous factors affect outcomes), some predictions have been made for various other disease states. For example, a change of 10% in the 6MWT was reported to represent the minimally important difference (with respect to respiratory outcomes) in patients with COPD (Alexandrou et al., 2021). Given the decline in cardiorespiratory health in those with advanced CKD, it would be expected that similar predictions would provide useful guidance for healthcare professionals supporting those with CKD (Hiraki et al., 2017).

Patients with CKD undergoing hemodialysis have been reported to have reduced activity levels, impaired mobility, and balance disturbance, which have been linked to an increase in falls risk (Shin et al., 2014). As an outcome metric, the sit‐to‐stand test evaluates the lower limb muscle strength, balance, and endurance (Table 1). Two out of nine articles in this systematic review measured sit‐to‐stand and both found a significant improvement in this outcome (Rosa et al., 2018; Zhang et al., 2020). Specifically, the study by Zhang and colleagues saw a reduction in time for participants to perform 10 sessions of sit‐to‐stand (Zhang et al., 2020). The study by Rosa and colleagues saw an increase in the number of sit‐to‐stand sessions performed by participants in a 30 s time limit compared to the control group (Rosa et al., 2018). Given the importance of strength and balance when moving from a seated to a standing position, efforts to improve this outcome can be a key factor in improving overall physical function and independence and reducing fall risk in dialysis patients (Wilkinson et al., 2019).

The timed up‐and‐go test has been well documented to have high validity and reliability to assess the overall mobility and functional ability in older adults and in CKD (de Castro et al., 2023; Ortega‐Pérez de Villar et al., 2018). Two articles included in this systematic review assessed timed up‐and‐go. The studies by Gadelha et al. (2021) and de Castro et al. (2023) found a significant improvement in timed up‐and‐go after resistance exercise intervention. Given the well documented validity of this test, future studies are necessary to truly identify the scope of benefit for resistance exercise in CKD and identify a clinically important difference in this test.

This systematic review aligns with findings from real‐world literature. In a randomized pilot trial conducted by Hiraki and colleagues, the impact of home‐based exercise therapy on kidney function in pre‐dialysis Stage 3–4 CKD patients was investigated (Hiraki et al., 2017). Participants were randomly assigned to either an exercise intervention (consisting of home‐based aerobic and resistance exercises) or a control group, with no significant baseline differences observed between the two (Hiraki et al., 2017). Notably, grip strength and knee extension muscle strength showed improvement solely in the exercise intervention group‐results that are similar to papers we see included in our systematic review (Hiraki et al., 2017).

Similar outcomes were observed in a single‐armed interventional study by Hamada and colleagues, wherein CKD patients underwent a 6‐month aerobic and resistance exercise intervention, with functional outcomes measured pre and post (Hamada et al., 2016). Significant improvements were noted in the 30‐s chair stand test (*p* < 0.001), single‐foot standing test (*p* = 0.001), and the 6‐min walk test (*p* = 0.02) compared to baseline measures (Hamada et al., 2016).

Contrarily, Cheema and colleagues reported no significant difference in the 6‐min walk test following 12 weeks of high‐intensity resistance exercise training during maintenance hemodialysis for end‐stage renal disease (Cheema et al., 2007).

Furthermore, Geenen and colleagues explored the impact of resistance exercise frequency on muscle function in stage‐3 CKD patients (Geneen et al., 2022). Twenty participants were assigned to either a low‐frequency exercises group (one session per week) or a high‐frequency group (three sessions per week) (Geneen et al., 2022). Both groups demonstrated significant improvements in sit‐to‐stand and the North Staffordshire Royal Infirmary (NSRI) walk test (involving walking for 50 m, climbing 22 steps, and walking back 50 m) compared to pre‐intervention measures (Geneen et al., 2022). The lack of significant differences in functional outcomes between the two intervention groups suggests that a lower frequency of resistance exercise could serve as a practical tool to enhance the overall quality of life for CKD patients.

Currently, there is a noticeable gap in the literature regarding the impact of exclusive resistance exercise on populations prone to developing sarcopenia, specifically those with CKD. Investigating the effects of whole body resistance exercise in Stage 4 and 5 CKD patients exclusively revealed promising outcomes, indicating that prescribing exercise interventions can improve functional capabilities. Furthermore, performing resistance exercise across different phases of dialysis (such as before or during dialysis), and varying frequency and intensity, underscores the adaptability of exercise interventions. This approach allows for tailored implementation based on patient preference and the clinical judgment of healthcare providers.

### 4.1 Limitations

Overall, participants included in the review were recruited from 3 countries and had relatively small sample sizes which limits the generalizability of the results, particularly as it relates to determining possible heterogeneity in exercise responses. Our search criteria focused on persons with Stage 4 or 5 CKD as these stages are when symptoms would begin to manifest and patients would be referred to a nephrologist (Vaidya & Aeddula, 2024). Furthermore, while we did not limit studies to those on dialysis, only studies with CKD patients receiving dialysis were found using our search strategy. Clearly, more studies are necessary to investigate the efficacy of resistance exercise in those with CKD prior to dialysis and those in earlier stages of kidney disease.

## 5 CONCLUSIONS

Physical performance in functional outcomes is one of the strongest predictors of future survival in dialysis patients (Chow et al., 2022). A few important conclusions can be drawn from this systematic review: (1) There is a dearth of information on resistance training in persons with Stage 4/5 CKD who are not undergoing dialysis. As dialysis is a well‐documented atrophic stimulus, clearly defining the benefits of whole‐body resistance training before those with CKD begin dialysis would be particularly important information for healthcare professionals and persons living with CKD. Does pre‐habilitation exercise improve functional outcomes for those on dialysis? Is dialytic atrophy mitigated in those who undertook whole body resistance exercise before beginning dialysis? (2) The evidence to date is not universally beneficial for CKD patients on dialysis with some metrics showing a benefit of resistance training and others showing no effect. We believe this lack of consistent benefit is likely due to the challenge of overcoming the atrophic stimulus of dialysis, as well as the physical disability caused by dialysis (balance disturbances, impaired mobility, etc.) limiting the ability of participants working out to their fullest extent. In conclusion, there were no adverse events reported with resistance training in this cohort and many studies show improvements in clinically important outcomes. Collectively, this systematic review presented evidence that whole‐body resistance training is generally associated with improved clinically‐relevant functional outcomes in stage 4 and 5 CKD patients undergoing dialysis. However, this systematic review also provide clear gaps for future study to allow those with CKD and their healthcare providers with important evidence‐based exercise recommendations.

### Implications for Future Research

With robust evidence from larger studies and concrete evidence, the integration of resistance exercise training into mainstream medical care for CKD patients can be implemented. This systematic review suggests that the prescription of whole‐body resistance training may be beneficial for those with stage 4 or 5 CKD and should be explored further. The data to date provides a promising foundation for the implementation of these larger studies.

## FUNDING INFORMATION

No funding sources were involved in this work.

## DISCLOSURE

The authors have nothing to disclose.

## ETHICS STATEMENT

All authors provided final approval of the version to be published. All people designated as authors qualify for authorship, and all those who qualify for authorship are listed. TJH is the guarantor of this work and, as such, had full access to all the data in the study and take responsibility for the integrity of data and the accuracy of the data analysis.

## Supporting information


Figure S1.

## Data Availability

Data generated or analyzed during this study are available from the corresponding author upon reasonable request.
