# Supplementary figures and images for: Whole body resistance training on functional outcomes of patients with Stage 4 or 5 chronic kidney disease: A systematic review
Source: Physiol Rep. 2024 Aug 12;12(15):e16151. doi: 10.14814/phy2.16151 (PMC11319065; doi:10.14814/phy2.16151)

776     **Supplementary Figure 1: Completed PRISMA Chart**

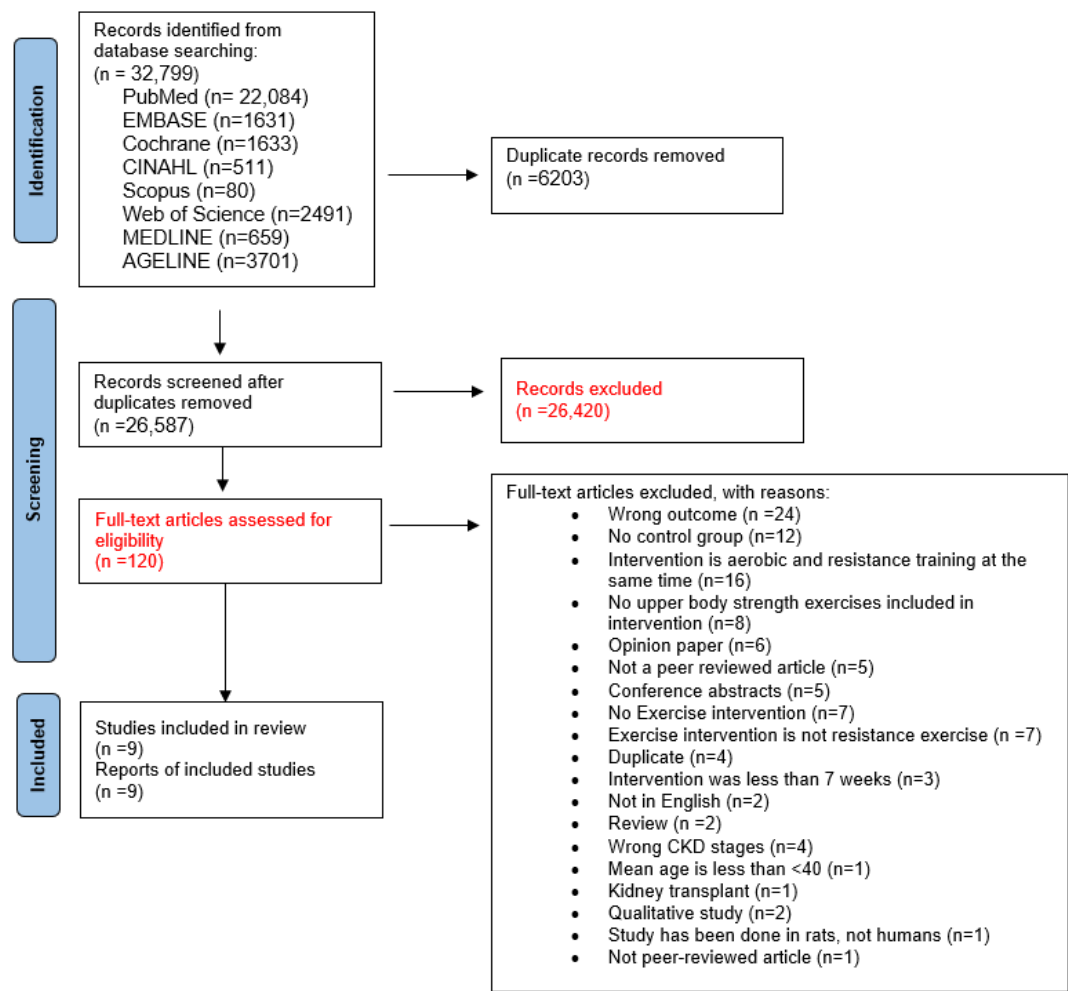

778     Note. Source: (Page et al., 2021).<sup>64</sup>

Supplement: Supplementary file 1 — Figure S1. [file PHY2-12-e16151-s001.pdf]
